# Temporal Regularity Detection and Rate Discrimination in Cochlear-Implant Listeners

**DOI:** 10.1007/s10162-016-0586-4

**Published:** 2016-09-29

**Authors:** Etienne Gaudrain, John M. Deeks, Robert P. Carlyon

**Affiliations:** 1 MRC Cognition and Brain Sciences Unit, 15 Chaucer Road, CB2 7EF Cambridge, UK; 2 CNRS UMR 5292, Lyon Neuroscience Research Center, Auditory Cognition and Psychoacoustics, Université Lyon 1, 50 av. Tony Garnier, 69366 Lyon Cedex 7, France; 3 Department of Otorhinolaryngology, University Medical Center Groningen-University of Groningen, Huispostcode BB20, PO Box 30.001, 9700 RB Groningen, Netherlands

**Keywords:** cochlear implant, pitch, temporal resolution

## Abstract

Cochlear implants (CIs) convey fundamental-frequency information using primarily temporal cues. However, temporal pitch perception in CI users is weak and, when measured using rate discrimination tasks, deteriorates markedly as the rate increases beyond 300 pulses-per-second. Rate pitch may be weak because the electrical stimulation of the surviving neural population of the implant recipient may not allow accurate coding of inter-pulse time intervals. If so, this phenomenon should prevent listeners from detecting when a pulse train is physically temporally jittered. Performance in a jitter detection task was compared to that in a rate-pitch discrimination task. Stimuli were delivered using direct stimulation in cochlear implants, on a mid-array and an apical electrode, and at two different rates (100 and 300 pps). Average performance on both tasks was worse at the higher pulse rate and did not depend on electrode. However, there was a large variability across and within listeners that did not correlate between the two tasks, suggesting that rate-pitch judgement and regularity detection are to some extent limited by task-specific processes. Simulations with filtered pulse trains presented to NH listeners yielded broadly similar results, except that, for the rate discrimination task, the difference between performance with 100- and 300-pps base rates was smaller than observed for CI users.

## Introduction

Poor pitch perception, and the consequent reduction in the ability to segregate competing sounds, remains one of the chief sources of disability for cochlear implant (CI) users. Because CIs convey fundamental-frequency (F0) information using purely temporal cues, a large number of studies have studied temporal pitch processing by CI users (Shannon 1983; Townshend et al. 1987; for a review, see Moore and Carlyon 2005). A common technique is to bypass the clinical speech processor and to require subjects to discriminate differences in the rate of an isochronous pulse train applied to a single electrode. Subjects can usually detect changes of a few percent at low pulse rates, but performance usually deteriorates markedly for rates higher than about 300 pps, although a minority of subjects can perform the task at somewhat higher rates (Hochmair-Desoyer et al. 1983; Kong and Carlyon 2010).

Although rate discrimination tasks undoubtedly measure processes that are important for pitch perception by CI users, they suffer from two limitations. First, it is not possible, with these tasks, to determine whether the rate-pitch perception deficit in CI listeners is due to peripheral or more central limitations. Because listeners perform the task by comparing the perceived pitches of two pulse trains, performance may be limited either by the way the sound is encoded in the auditory periphery and/or by more central mechanisms that convert this neural activity into a representation of pitch. This means that additional methods must be employed if one is to better identify the neural basis for poor pitch perception by CI users. Second, rate discrimination tasks do not require the listener to estimate the inter-pulse intervals in a stimulus with a high degree of temporal accuracy; it is instead possible to perform the task by counting the number of pulses in the time interval between the first and last pulse (Carlyon 1997). Hence, it is possible to perform the task without necessarily encoding the fine temporal intervals that are, presumably, necessary for more demanding pitch tasks such as the estimation of musical intervals.

The present study compares performance on a rate discrimination task with that on a different task, temporal jitter detection, that requires fine temporal processing and that does not require the listener to compare the pitch values of different sounds. Both tasks were performed at two different baseline rates and for stimuli applied to two different electrodes. In order to determine whether the two tasks share a common limitation, we investigated whether they showed a similar effect of base pulse rate, and whether the variation in performance across listeners and conditions (place and rate of stimulation) was the same for both tasks. In addition, we performed manipulations with bandpass filtered pulse trains presented to normal hearing (NH) listeners, in order to assess whether the observed limitations in performance were specific to electrical stimulation of deafened ears. These filtered pulse trains are analogous to electric pulse trains presented to CI listeners, in that they present temporal information to auditory nerve fibres tuned to higher frequencies than the repetition rate, and do not provide any place-of-excitation cues (McKay and Carlyon 1999; van Wieringen et al. 2003).

## Methods

### Participants

Five CI users (aged 60 to 81) and seven NH listeners (aged 18 to 35) took part. All the CI users were recipients of the Nucleus device (Cochlear Corp., Melbourne, Australia); their details are provided in Table 1. All of the NH listeners had audiometric thresholds below 20 dB HL at 1, 2, 4, 8 and 10 kHz. All subjects provided written informed consent and received a financial compensation for their participation. The study was approved by national and local Ethics Committees.

**Table 1:** Details of the five CI users who participated in the experiment

	Implanted ear	Implant type	Implant use duration (years)	Deafness duration (years)	Aetiology
C01	L	CI24M	>2	10	Chronic suppurative otitis media
C02	L	CI24M	10	5	Progressive unknown
C03	R	CI24M	>2	22	Otosclerosis/noise induced
C04	L	CI24M	11	>10	Progressive unknown
C05	L	Freedom	2	15	Congenital, progressive

### Tasks and Stimuli

The two tasks, jitter detection and rate discrimination, both used pulse trains that were presented electrically to CI listeners and acoustically to NH listeners. Below are given the details of the pulse train generation, i.e. how the time intervals were constructed for each task. The details of electrical and acoustical stimulation are given in the following section “Procedure and Apparatus”.

In both tasks, two rate conditions were used: 100 and 300 pps, representing the average rate of the pulse train in the standard (non-signal) stimulus. Two place conditions were also used: the pulse train was set to stimulate either a more apical region or a more basal region (see “Procedure and Apparatus” for details).

#### Jitter Detection Task

**FIG. 1:**
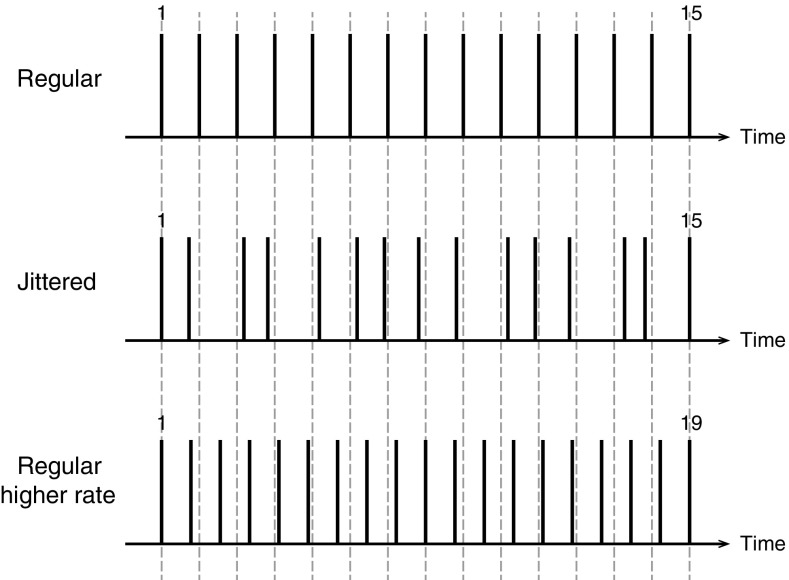
Schematic representation of the different stimuli used for jitter discrimination (*middle panel*) and rate discrimination (*lower panel*).

The jittered stimuli were created by randomizing the duration of the intervals between pulses (see Fig. 1). The interval durations were first picked from a normal distribution centred on the mean duration corresponding to the reciprocal of the average pulse rate (10 or 3.33 ms). To make the stimuli more or less jittered, the width of the distribution was varied: standard deviations of 7 to 45 % of the mean interval duration were used. To avoid overlap between intervals (because normal distributions go from minus infinity to plus infinity), all intervals were constrained to deviate by no more than 1 standard deviation from the average. Finally all intervals were scaled so that the duration of the jittered stimulus was exactly the same as that of the regular stimulus.

#### Rate Discrimination Task

For this task, the duration was kept exactly fixed and so increases in pulse rate were accompanied by increases in the number of pulses presented. The standard had 15 pulses, while the test stimuli had 16 to 21 pulses, yielding rate increases from 7 to 43 %.

### Procedure and Apparatus

For both groups, the task on each trial was to determine which of the second or third interval was most different from the two others (3I-2AFC). Each condition was repeated 50 times, yielding a total of 6 steps × 4 conditions × 50 repetitions = 1200 trials for each of the tasks. On each trial, a standard stimulus (regular, at base rate 100 or 300 pps) was presented, followed by the same stimulus and a test stimulus in a random order. Buttons were lit up on the screen as the sounds were played. The participants then had to click on the button corresponding to the sound they thought was different from the two others (either the second or the third one). The correct answer flashed on the screen after each response. The level of the three sounds was roved by 4 current units (about 0.68 dB) below comfort level (for the CI group) or by 5 dB (for the NH group) and the participants were instructed to ignore loudness as a possible cue.

#### Cochlear-Implant Group

For the CI group, the experiment was broken up in sessions of 3 h. Participants all started with the jitter detection task at 100 pps on electrode #11, followed by jitter detection at 300 pps on the same electrode. On the second session, they performed the rate discrimination task on this same electrode, at the two base rates. On the third and fourth session, the experiment was repeated on electrode #17 (or #18). For some participants, more sessions were necessary to reach the end of the experiment.

Direct stimulation of the implant was performed using an L34 programmable processor (Cochlear Corp.). The experiment was programmed in Matlab (The MathWorks), using custom software (“PyNIC”) that functioned as a connector to the Python interface of the NIC2 drivers (Cochlear Corp.). Pulse trains consisted of anodic-leading biphasic pulses presented in monopolar (MP1 + 2) mode, with a phase duration of 40 μs and an inter-phase gap of 8 μs. For the “basal” condition, electrode #11, in the middle of the array, was used. For the “apical” condition, four of the patients were tested on electrode #17. The fifth patient was tested on electrode #18 instead, because electrode #16 was switched off in their map.

All stimuli were loudness balanced at each stimulation site and base rate. To loudness balance two stimuli, A and B, the most comfortable level (MCL) of A was first measured by asking the subject to rate loudness on an 11-point clinical loudness scale, where MCL corresponded to point 7. Stimuli A and B were then presented successively, with the level of A being MCL, and that of B being initially a random value chosen to be safely lower than MCL. The participant then had to adjust the level of B to match that of A by pressing one of six buttons showing 1 to 3 “−” or “+” signs, which corresponded to 1, 2 and 5 current units decrease or increase. Once the participants were satisfied with the level adjustment, they clicked on a button labelled “OK”. The roles of A and B were then inverted, and the procedure was started over. The average difference between the levels of A and B across procedures was used as the loudness balancing correction for this stimulus pair. In the jitter task, threshold, comfort level and maximum comfortable level were measured for the regular, 20 %-jitter and 45 %-jitter stimuli. As these levels were found to be the same or very close to each other, loudness balancing was only performed between the regular and the 45 %-jitter stimuli, with values for all other jitter amounts being linearly interpolated. In the rate discrimination task, the stimuli with 18 pulses (21 %) and 21 pulses (43 %) were both loudness-balanced against the standard 15 pulses stimulus. The current level difference yielding equal loudness for the other number of pulses was interpolated from these measurements.

#### Normal-Hearing Group

For the NH group, the same 3I-2AFC task was used with the same graphical user interface. However, instead of performing the loudness balancing procedure, all stimuli were presented at the same rms level of 67 dB sound-pressure level (SPL), which was roved by a total of 5 dB for each presentation. The participants sat in a double-walled sound attenuated booth. The sounds were presented using Matlab, through an ASUS Xonar Essence STX soundcard, a TDT PA4 attenuator, a TDT HB7 headphone buffer, and Sennheiser HD 650 headphones. Stimulus levels were checked using a KEMAR Type 45DA head assembly. Trials with each of the two frequency regions were mixed together in a single block. Participants performed the jitter detection task in a first 2-h session and came back a week later to do the rate discrimination task in a second session.

The acoustic stimuli consisted of monophasic pulses. The stimuli were sampled at 44,100 Hz, 16 bits, and the pulses were one-sample wide. The inter-pulse intervals were rounded off to the nearest sample, and the resulting pulse train was then filtered in a specific frequency band. The “apical” and “basal” conditions were implemented using two frequency regions, roughly analogous to the use of two electrodes in the CI group: a HIGH region [3900, 5400] Hz and a VERY-HIGH region [7800, 10,800] Hz (8th order Butterworth). At the higher base rate, the lower boundary of the HIGH region is located on the 13th harmonic. The harmonic structure was therefore most likely to be unresolved for both base rates (100 and 300 pps) in both frequency regions. In each trial, a low-pass filtered (4th order Butterworth, and the 3-dB cutoff was the lower frequency of the frequency band of the pulse train) “threshold equalizing noise” (TEN; Moore et al. 2000) was played simultaneously with the filtered pulse trains. The noise started 750 ms before the first pulse train, with a 525-ms raised cosine ramp, and ended 750 ms after the last pulse train with a symmetric ramp. The noise level at 1 kHz was set to 15 dB below the spectrum level of the filtered pulse train in its passband.

In addition, following piloting, the range of jitter values was extended downwards to 4 % for the NH participants, compared to 7 % for the CI users.

### Statistical Analyses

All statistics were performed using R 3.2.3 (R Core Team 2015). All percent-correct scores were transformed into rationalized-arcsine-units (RAU; Studebaker 1985) to reduce heteroscedasticity problems related to the use of the percent-scale. Main effects of and interactions between variables were primarily analysed using analyses of variance (ANOVA) on general linear models (GLM) adapted for repeated measures (using the “ez” package, v4.3, Lawrence 2015). These are referred to as “ANOVA” in the following section. The Greenhouse-Geisser correction for lack of sphericity was applied when necessary. When this was the case, non-integer degrees-of-freedom are reported with the *F* value. Reported effect sizes are calculated according to Bakeman (2005). All reported *t* tests are two-tailed Welch two-sample comparisons. It was not possible to use an ANOVA to compare performance between CI and NH users for the jitter task because the values of jitter used were not the same for the two tasks. Therefore between-group comparisons were assessed using a linear mixed model (LMM). The model was fitted using the “lme4” R-package, v1.1.11 (Bates et al. 2015) and *p* values were calculated using the Satterthwaite approximation of the “lmerTest” R-package, v2.0.30 (Kuznetsova et al. 2016).

When correlating performance between two tasks, it is important to distinguish between two sources of variance (Bland and Altman 1995a, b). *Inter-subject* variability reflects the fact that some subjects may perform better than others on all tasks. It could be due to differences in peripheral neural survival, to higher-order auditory processes, or to non-sensory factors including the ability to maintain attention to a boring task. It is of less interest but was assessed by averaging performance across conditions for each subject and task and then calculating the correlation. *Intra-subject* variance refers here to whether the pattern of results for a given subject on one task also occurs for the other task. For example, if a subject is better at rate discrimination in the apical region than in the basal region, is this also true for that subject in the jitter task? To test this, we entered the scores on the jitter task into an analysis of variance (using the “car” package, v2.1.1, Fox and Weisberg 2011), with subject as a fixed factor and the rate discrimination scores as a co-variate. The correlation coefficient is then equal to the ratio of the sum of squares accounted for by the co-variate to the total of that value plus the corresponding error variance (Bland and Altman 1995a). However, it is important to rule out effects that are common to the group as a whole. For example if, as was indeed the case, both tasks showed better performance at 100 pps than at 300 pps, this could lead to an apparently significant correlation. For this reason, where significant correlations were obtained, the analysis was repeated with base pulse rate entered as a fixed factor in order to partial-out this effect.

## Results

### Cochlear-Implant Subjects

#### Overall Effects of Base Pulse Rate and Place of Stimulation on Performance in the Rate and Jitter Tasks

**FIG. 2:**
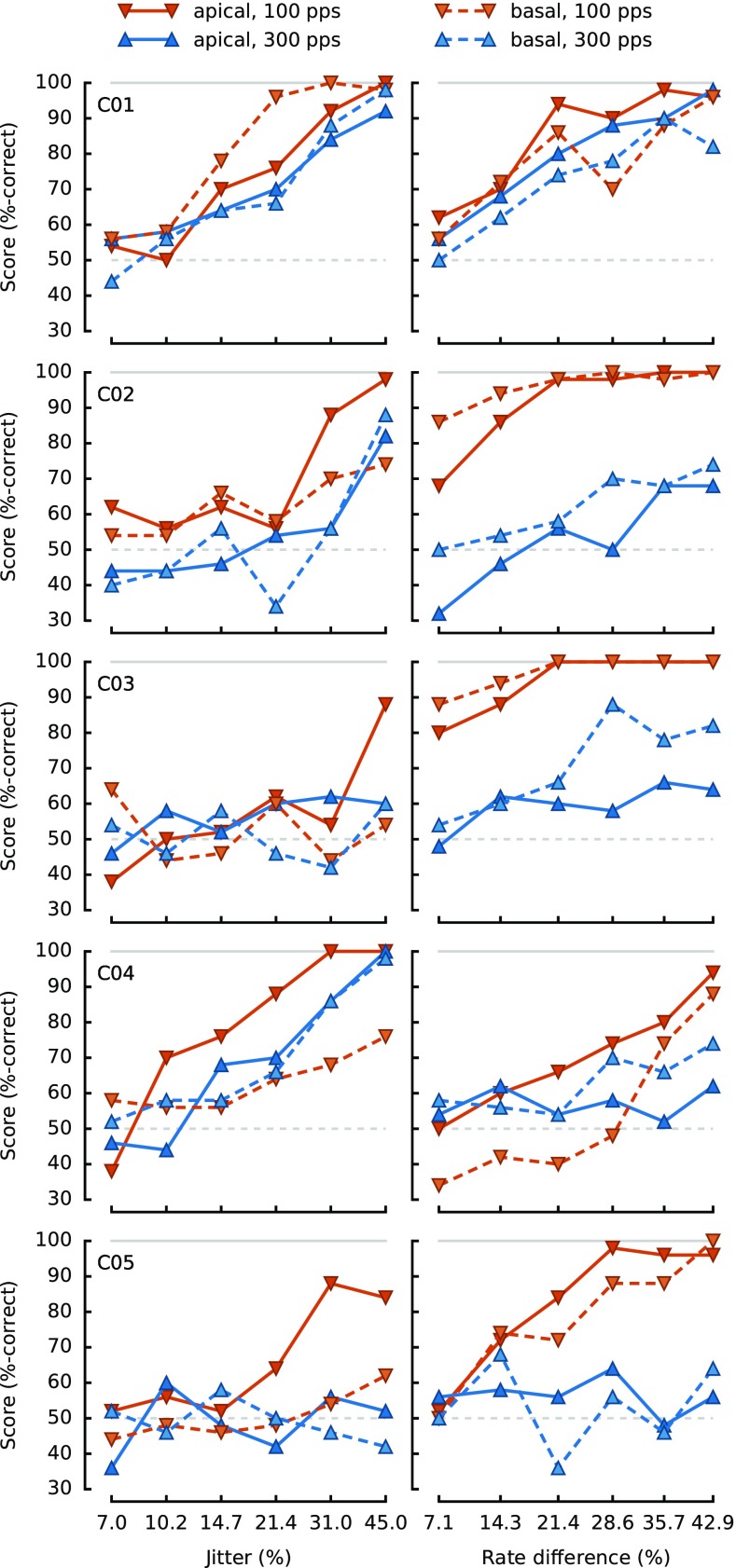
Individual CI data for jitter detection (*left column*) and rate discrimination (*right column*). The lower base rate (100 pps) is marked with *downward pointing triangles* (*red*), while the higher base rate (300 pps) is marked with *upward pointing triangles* (*blue*). The apical excitation site is traced with a *solid line*, while the basal excitation site is traced with a *dashed line*.

**FIG. 3:**
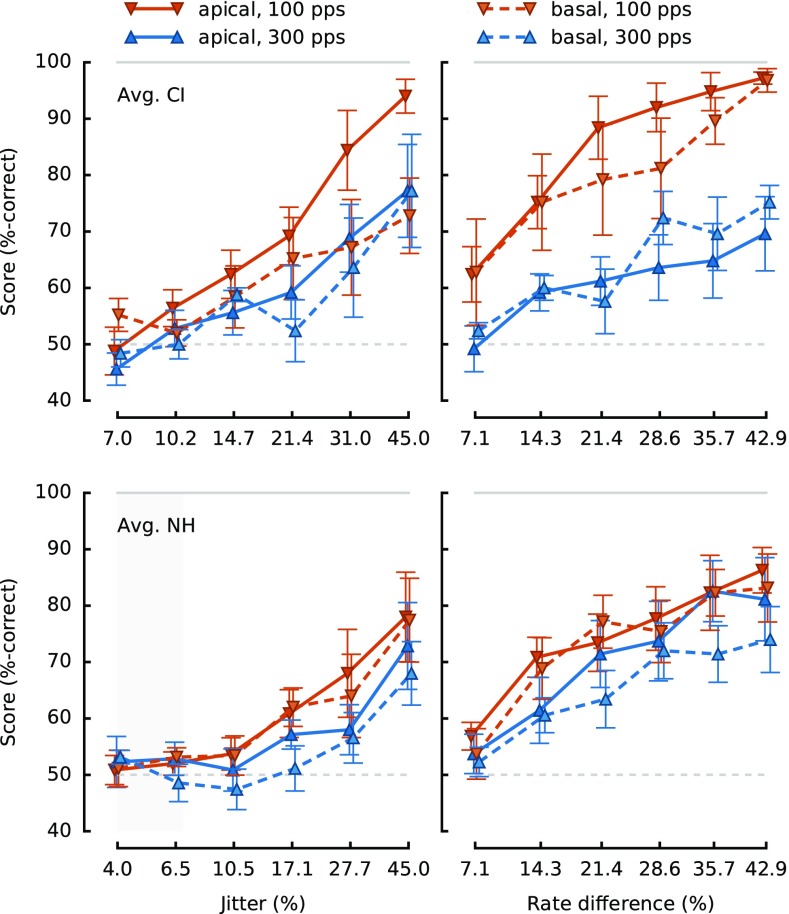
Average jitter detection (*left column*) and rate discrimination (*right column*) data for CI (*top row*) and NH (*bottom row*) listeners. Details are the same as for Figure 2. For NH listeners, “apical” corresponds to the HIGH region while “basal” corresponds to the VERY-HIGH region.

Individual results are shown in Figure 2, and the average across the five subjects is shown in Figure 3. Separate ANOVAs were performed on the RAU scores for each task.

For the rate discrimination task, there were significant effects of the base rate [*F*_(1,4)_ = 8.41, *p* = 0.044, *η* ^2^_*p*_ = 0.42] and of the rate difference [*F*_(5,20)_ = 28.08, *p* < 0.001, *η* ^2^_*p*_ = 0.36], but not the electrode [*F*_(1,4)_ = 0.05, *p* = 0.83, *η* ^2^_*p*_ < 0.01]. None of the interactions were significant [*p* > 0.066].

Our results are consistent with a wide body of literature showing that rate discrimination deteriorates with increasing base rate (Shannon 1983; Townshend et al. 1987; Kong and Carlyon 2010), and with evidence that, for monopolar stimulation, there is no consistent effect of the place of stimulation (Baumann and Nobbe 2004). It is however possible that a place effect might have been observed if we had tested the most apical electrode of the (longer) array used by MedEl Ltd., as has been recently observed by Stahl et al. (2013).

For the jitter task, the base rate also had a significant effect [*F*_(1,4)_ = 9.31, *p* = 0.038, *η* ^2^_*p*_ = 0.06], with performance being worse at 300 pps than at 100 pps. Similarly to the rate task, the place of stimulation (electrode) did not have a significant effect [*F*_(1,4)_ = 3.42, *p* = 0.14, *η* ^2^_*p*_ = 0.03]. Performance increased, as expected, with increasing amounts of jitter [*F*_(5,20)_ = 10.66, *p* < 0.001, *η* ^2^_*p*_ = 0.43]. None of the two-way interactions were significant [*p* > 0.11], and the three-way interaction was only a trend [*F*_(2.5,9.9)_ = 3.47, *p* = 0.065, *η* ^2^_*p*_ = 0.04].

The results show a similar pattern of performance for the two tasks, with an effect of base rate but not of which electrode is stimulated. This shows that the “high rate limitation”, previously observed for rate discrimination tasks, also occurs for a monaural task that does not require the comparison of two different pitch values. To test whether there was a more subtle difference in the way base rate affected performance in the two tasks, the data were combined into a four-way “task × electrode × base rate × change amount” ANOVA. Because the “change amounts” corresponded to different manipulations in the two tasks, it is not appropriate to consider the main effect of task or the interaction of any effect with the amount of change. However, it is appropriate to examine the interaction between task and base rate. This was not significant [*F*_(1,4)_ = 4.45, *p* = 0.103, *η* ^2^_*p*_ = 0.092].

#### Correlations Within and Between Subjects

The above analyses show that, at least to a first approximation, the two tasks are similarly affected by the physical characteristics of the stimulus. This is consistent with, but does not prove, the idea that the limitations in the two tasks share a common locus. However, it is known that CI users vary in their ability to perform the rate discrimination task, and that the effect of both base rate and place of stimulation can vary markedly across subjects. It is therefore worthwhile to consider whether a similar pattern of variation occurs for the two tasks. As described in the “Methods” section, we consider inter- and intra-subjects sources of variation separately.

**FIG. 4:**
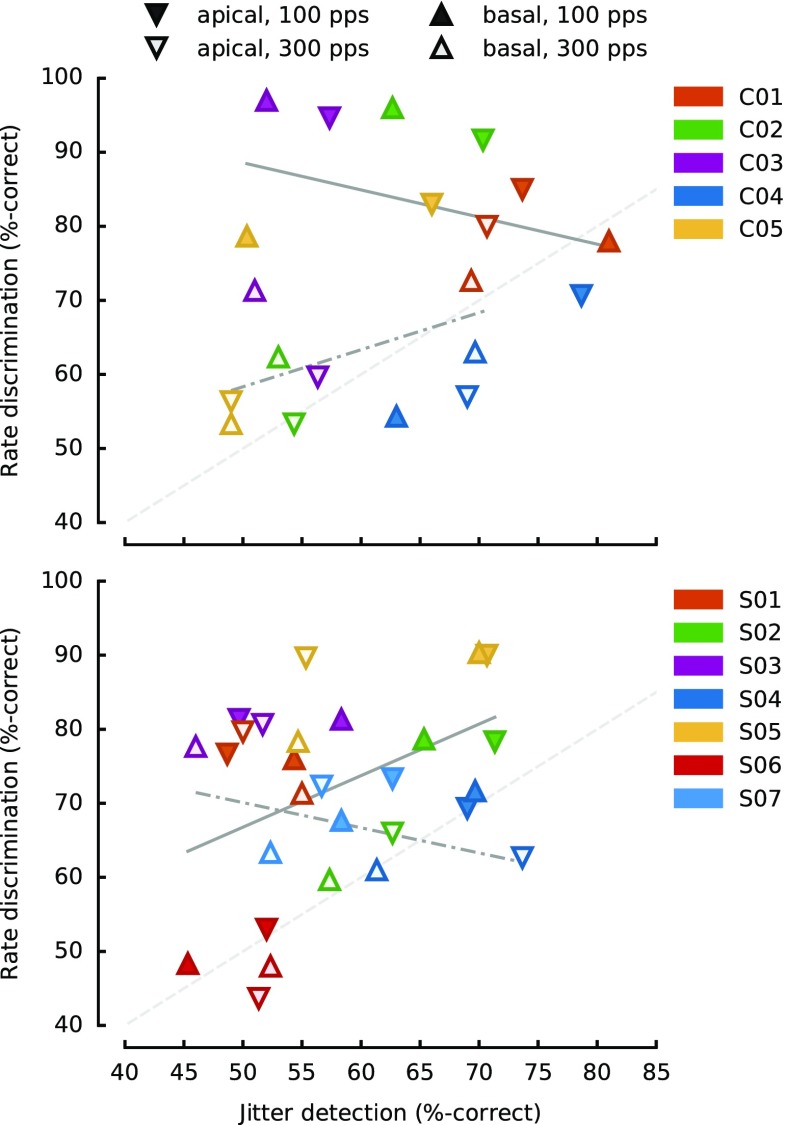
Correlations between jitter detection and rate discrimination for CI (*top panel*) and NH (*lower panel*) listeners. Unlike in Figures 2 and 3, the *downward pointing symbols* represent the apical site, while the *upward pointing symbols* represent the basal site. The *opened symbols* correspond to the 300-pps base rate, while the *filled symbols* correspond to the 100-pps base rate. Each participant is plotted with a different colour. The *grey lines* show the regression lines for the 100-pps base rate (*solid*) and for the 300-pps base rate (*dot-dashed*).

The inter-subject correlation was not significant [*r* = −0.29, *p* = 0.64]. This reflects the fact that, over the range of values tested here, some subjects (C03 and C05) performed better on the rate task than on the jitter task, whereas the opposite was true for others (e.g. C04). This can be seen both by inspection of Figure 2, and in Figure 4A where the scores for each condition (averaged across the amount of change) are shown, with each subject represented by a different colour.

Figure 2 also shows that the pattern of results for individual subjects could also differ substantially between the two tasks. Consider the effect of electrode at the lower rate, where performance was above chance for all subjects (solid vs. dotted red lines in Fig. 2). The effect of electrode on performance was in the opposite direction for the two tasks for subjects C01, C02 and C03, and in the same direction for C04 and C05. Yet the intra-subject correlation just reached significance [*r* = 0.52, *p* = 0.04], but dropped below significance level [*r* = 0.16, *p* = 0.57] once the common effect of base rate was removed (see “Methods”). It is appreciated that this non-significant effect might be due to a lack of power, given the small number of subjects tested. However, the design was sufficiently sensitive to reveal a correlation in a single task, jitter detection, between the two rates [*r* = 0.88, *p* = 0.049], despite the small number of participants. In addition, when we obtained the intra-subject correlation between the average of the odd- vs. even-numbered trials for each condition, these were highly significant for both tasks [jitter: *r* = 0.85, *p* < 0.001; rate: *r* = 0.96, *p* < 0.001].

### Normal-Hearing Subjects

#### Overall Effects of Base Pulse Rate and Place of Stimulation on Performance in the Rate and Jitter Tasks

Average results for the NH listeners are plotted in Figure 3B. For jitter detection, only the amount of jitter had a significant effect [*F*_(1.3,7.6)_ = 7.32, *p* = 0.024, *η* ^2^_*p*_ = 0.32], while the spectral region [*F*_(1,6)_ = 1.44, *p* = 0.28, *η* ^2^_*p*_ = 0.005] and the base rate [*F*_(1,6)_ = 4.09, *p* = 0.09, *η* ^2^_*p*_ = 0.04] did not. None of the interactions were significant [*p* > 0.19].

For rate discrimination, higher scores were observed in the HIGH region (“apical”, solid lines; 73.5 RAU) than in the VERY-HIGH region (“basal”, dashed lines; 69.8 RAU) [*F*_(1,6)_ = 8.62, *p* = 0.026, *η* ^2^_*p*_ = 0.02]. The base rate also had a significant effect [*F*_(1,6)_ = 8.64, *p* = 0.026, *η* ^2^_*p*_ = 0.04] with higher scores at 100 pps (74.9 RAU) than at 300 pps (68.5 RAU). Finally, while the rate difference also had a significant effect on the scores [*F*_(5,30)_ = 22.51, *p* < 0.001, *η* ^2^_*p*_ = 0.32], none of the interactions were significant [*p* > 0.10].

The results show that, for the NH listeners, the two tasks differed in terms of which effects reached statistical significance. Specifically, the effects of place of excitation and of base rate reached significance for the rate task but not for the jitter task. However, the relevant question is not whether the sizes of these two effects fall either side of an arbitrary (albeit widely adopted) significance level of 5 %, but whether they differed significantly from each other. We therefore combined the results from the two tasks into “task × spectral region × base rate × change amount” ANOVA. As noted in our discussion of the CI results, it is not appropriate to consider the main effect of task or the interaction of any effect with the amount of change. However, it is appropriate to examine the interaction between task and base rate and between task and spectral region. Neither of these interactions were significant, and so there is no evidence that, on average, the effects of base rate or frequency region differ between the two tasks [task × base rate: *F*_(1,6)_ = 0.22, *p* = 0.65, *η* ^2^_*p*_ < 0.001; task × spectral region: *F*_(1,6)_ = 0.76, *p* = 0.42, *η* ^2^_*p*_ = 0.001].

#### Correlations Within and Between Subjects

Correlation analyses were performed, but only the jitter amounts from 6.5 to 45 % were included, for consistency with the data from the CI participants. As for the CI users, inter-subject correlation were not significant [*r* = 0.22, *p* = 0.64]. The intra-subject correlation was significant [*r* = 0.58, *p* = 0.004], but this effect disappeared once we removed the common effect of base rate [*r* = 0.40, *p* = 0.08]. Partialling out the spectral-region factor in addition to base rate further reduced the correlation [*r* = 0.30, *p* = 0.21]. Hence, as for the CI participants, we could find no evidence for a correlation between the two tasks beyond the common effects of base rate and spectral region.

### Comparison of NH and CI Listeners

#### Jitter Detection

Because the jitter values were not the same for the two groups, we performed a LMM instead of an ANOVA. The fixed factors were group (CI or NH), jitter (as a continuous variable), base rate and place (apical or basal electrode for the CI, and HIGH or VERY-HIGH for the NH). The only random factor was subject, in the form of a random intercept.

Only jitter had a significant main effect on the scores [*F*_(1,276)_ = 66.7, *p* < 0.001]; group, base rate and place did not [*p* > 0.17]. Here we focus on interactions involving the group factor, to determine whether the effect on performance of any of the other parameters differed between NH and CI listeners. The group factor did not interact significantly either with the place of stimulation or the base rate. The amount of jitter did interact with group, reflecting a slightly steeper increase in performance with increasing jitter for the CI compared to the NH listeners [*F*_(1,276)_ = 6.65, *p* = 0.010]. There was also a three-way jitter × group × place interaction [*F*_(1,276)_ = 7.79, *p* = 0.006] and the four-way interaction group × jitter × place × base rate was also significant [*F*_(1,276)_ = 4.65, *p* = 0.032]. Both of these effects reflect the especially steep slope of the psychometric function for the CI listeners in the apical 100-pps condition (solid red line, Fig. 3, top-left panel).

To compare the two groups more directly, and eliminate the jitter variable, it is possible to extract thresholds from the individual data by fitting cumulative Gaussian functions to the data. One inconvenience is that when subjects were not able to perform the task at all (in a specific condition), the fitting procedure fails and there is no threshold available for this participant in this condition. The comparison between CI and NH can therefore only be performed in the most apical condition (electrode #17 or #18, or HIGH region), at the base rate of 100 pps, where most participants had a measurable threshold (all except S01 and S06 from the NH group). The CI listeners reached 75 % correct for 26 % jitter (s.e. 4.2 percentage points). The NH listeners needed 25 % jitter to achieve the same level of performance (s.e. 3.3 percentage points), a difference that was not significant [*t*_(7.6)_ = 0.13, *p* = 0.90]. Overall, then, results for the jitter task were broadly similar for the two groups, both in terms of co-variation with other parameters and in the overall level of performance.

#### Rate Discrimination

The same method can be applied to the rate discrimination data. We first used the same LMM as for the jitter data, but applied to the rate discrimination RAU scores: rate difference is now the regression factor, while group, place and base rate are all binary factors. This analysis showed a significant effect of the rate difference [*F*_(1,276)_ = 60.0, *p* < 0.001]. There was also a rate difference × group interaction [*F*_(1,276)_ = 6.37, *p* = 0.012], showing that the slopes of the psychometric functions differed between NH and CI listeners. Importantly, the three-way rate difference × group × base rate interaction was significant [*F*_(1,276)_ = 7.62, *p* = 0.006], suggesting that the effect of rate on the psychometric functions was different between the two groups. Inspection of Figure 3 suggests that these statistical outcomes were due to the effect of base rate being greater for the CI than for the NH listeners. This can first be seen as a change in slope of the rate difference effect: the slopes of the effect of rate difference^1^ changed, in NH participants, from 0.91 to 0.69 RAU/percentage point of rate difference when changing the base rate from 100 to 300 Hz, while for the CI participants, it fell from 1.14 to 0.55 RAU/percentage point. This effect also shows in the average scores for each condition and group: scores decreased by 26 RAU from 100 to 300 pps in CI users, while they only decreased by 6 RAU in NH listeners. Finally, the LMM revealed no main effect of group [*F*_(1,86)_ = 0.39, *p* = 0.53] and no main effect of base rate [*F*_(1,276)_ = 3.29, *p* = 0.07] or place [*F*_(1,276)_ = 0.024, *p* = 0.88]. All the other interactions were also non-significant [*p* > 0.22].

Average discrimination thresholds, at 100 pps, in the more apical region, were again obtained (except for S06) and were again found to be not significantly different for CI (15 %, s.e. 4.0 percentage points) than for NH (21 %, s.e. 3.1 percentage points) [*t*_(7.9)_ = −1.21, *p* = 0.26]. In summary, the two groups showed roughly the same overall level of performance on the rate discrimination task, but the CI group showed a stronger dependence on base rate.

#### Correlation Between Jitter Detection and Rate Discrimination

Because no group main effect was observed in the two tasks, it was possible to pool the subjects from the two groups into a single correlation analysis in order to increase statistical power. Moreover, if, as suggested by the correlation analyses performed within each group, the two tasks show some degree of independence, it should remain the case when assessing this independence across subject groups.

Taking the subjects of the two groups, and doing the same correlation analyses as previously presented in each of the groups separately, we obtained very similar results. To compute the average scores in the jitter task, we only kept the scores for jitter values ≥7 % for the NH, in order to make them comparable to the CI data. The inter-subject correlation was not significant [*r* = 0.08, *p* = 0.81] but the intra-subject correlation was [*r* = 0.52, *p* = 0.001]. Once more, partialling out base rate, the correlation disappeared [*r* = 0.21, *p* = 0.21], which again indicates no correlation between the ability to detect jitter and to discriminate rates beyond the common dependence on base rate and place.

## Discussion

### Comparison to Previous Results

#### Rate Discrimination

Our results are consistent with several well-established findings on rate discrimination. All CI subjects could perform the task with a base rate of 100 pps, but performance dropped markedly at the 300-pps rate, where performance was close to chance for several subjects and conditions. No overall difference was observed between the two electrodes tested (Baumann and Nobbe 2004), and, for both electrodes, the rate difference corresponding to 71 % correct was about 10 % of the standard rate. This is close to the average threshold of 7.3 % (range 2–16 %) calculated by Moore and Carlyon (2005) from a sample of 19 subjects taken from five studies. For our NH subjects, the threshold for a 100-pps pulse train was about 20 %, but not significantly different from the CI users. Finally, the fact that performance dropped more markedly with increasing base rate in the CI listeners, compared to NH listeners, is consistent with the observation that the “upper limit” of rate pitch is usually lower in CI than in NH listeners (Carlyon and Deeks 2002; Kong and Carlyon 2010).

The rate discrimination limen (DL) of 20 % observed here for 100-pps pulse trains is higher than the value of about 4 % reported by several authors for unresolved pulse trains of a similar rate (Shackleton and Carlyon 1994; Krumbholz et al. 2000; Carlyon and Deeks 2002). One reason for this may be our combination of a 5-dB level rove and an odd-man-out procedure. Although subjects were instructed to ignore loudness cues, it is nevertheless the case that, on some trials, the “most different” stimulus would not have had the standard rather than the signal rate, if that stimulus happened to have an extreme value of the level rove. This effect may be larger than in a two-interval task, where the subject is required to make a judgement based on pitch. In this regard, it is worth noting that large effects of a level rove have been observed for CI users in an odd-man-out task; Baumann and Nobbe (2004) found that rate DLs for a 200-pps pulse train increased from about 15 % to about 40 % as the amount of roving increased from zero to 10 % of the dynamic range. This may have been due to a “distracting” effect of the level rove and the effects of level on pitch in CI users (Carlyon et al. 2010). Note that, in the present study, the same level rove was applied in all tasks and conditions, and so although the rove may have decreased performance overall, it is unlikely to have affected the pattern of results.

#### Jitter Discrimination

Our CI listeners’ discrimination thresholds were, on average, approximately 25 and 35 % at base rates of 100 and 300 pps, respectively. These are slightly higher than those obtained for two CI listeners by Dobie and Dillier (1985), whose thresholds at 125 and 250 pps were 16 and 19 % for one listener and 19 and 33 % for the other listener.

Thresholds for our NH listeners were broadly similar to those obtained for CI listeners. This contrasts with the results of Dobie and Dillier (1985) who reported jitter detection thresholds for NH listeners that were about 1 %, more than 10 times smaller than for their CI listeners and markedly smaller than those for our NH listeners. However, it should be noted that they used unfiltered click trains in NH, meaning that some of the harmonics would have been resolved by the peripheral auditory system, thereby making place-of-excitation cues available. In the present study, the pulse trains were filtered in high frequency regions to prevent resolvability of the harmonics, and potential distortion products were masked with a noise. Tsuzaki and Patterson (1998) reported an average detection threshold of 8 % for a base rate of 100 pps, considerably lower than the threshold of 25 % observed here for pulse trains filtered into the HIGH region. Tsuzaki and Patterson did high-pass filter the click trains above 1.6 kHz, thus removing spectral cues related to resolvability of the harmonics. However, they did not use a low-pass noise to mask distortion products, which may therefore have provided an additional cue. In addition, as noted above, the level rove used in the present study may have increased the DL.

### A Common Limit to Temporal Processing?

Broadly speaking, CI listeners’ performance on our two tasks varied in a similar fashion with the different stimulus parameters, with no effect of electrode and with performance being better at 100 pps than at 300 pps. This latter finding suggests that the deterioration in temporal processing at high overall rates, which is well-established for rate discrimination, can also be observed in a monaural task that does not require listeners to make an explicit judgement of pitch. One *caveat* to this conclusion comes from the observation that both of Dobie and Dillier’s CI subjects showed that, as the base rate was increased up to very high rates, jitter detection threshold dropped from an average of 24 % at 250 pps to 10 % at 1000 pps. It may be that, for the jitter task, additional cues are available at high pulse rates, as discussed below.

Further evidence that different cues may at least partially underlie performance in the two tasks comes from our finding that there was no inter- or intra-subject correlation between rate and jitter discrimination scores. This contrasts with the results for ITD discrimination of pulse trains presented to bilaterally implanted listeners, which is also known to deteriorate markedly as pulse rate increases beyond 100–200 pps. Recently, Ihlefeld et al. (2015) measured rate discrimination at three electrodes (apical, mid-array and basal) in each ear of eight bilateral CI users, and compared the results to ITD discrimination for pulse trains presented to each of the three pitch-matched pairs. Those measures were obtained at pulse rates between 100 and 500 pps, with standards and signals that differed by 35 % for the rate task and 500 μs for the ITD task. Ihlefeld et al. observed a correlation between ITD discrimination and rate discrimination on the worse of the two pitch-matched electrodes; this correlation remained significant even when the main effects of rate, electrode position and subject were excluded.

Taken together, the above results are consistent with a scenario whereby there is a limit to high-rate temporal processing that is common to rate discrimination, jitter discrimination and ITD discrimination tasks, but where some different cues may influence performance on the three tasks. One possibility is that, with irregular stimuli, the neural response may become amplitude modulated for one of two reasons. First, refractory effects may cause the neural response to become smaller after short inter-pulse intervals as compared to long ones, as has been observed in the electrically evoked compound action potential (ECAP) by Carlyon and Deeks (2013). Conversely, very short inter-pulse intervals may result in a single, high-amplitude response, due to facilitative effects in the auditory nerve and/or more centrally along the auditory pathway (Hancock et al. 2012). In order to test this hypothesis, we measured performance on the jitter detection task for 100- and 200-pps pulse trains presented to the basal/apical electrode of subject C01, with the amplitude of each pulse either constant or roved by +/− 4 CU from pulse to pulse. Performance was unaffected by the level rove, suggesting that, at these rates, performance was not mediated by the detection of amplitude modulation in the neural response. However, we think it is highly likely that such cues will come into play at higher rates, given Dobie and Dillier’s finding that listeners could detect 10 % of jitter at 1000 pps, where it is unlikely that fine temporal cues are preserved in the auditory periphery. In this regard, it is worth noting that ITD detection at high rates can be improved by jittering the pulses synchronously across the two ears (Laback and Majdak 2008). Hancock et al. (2012) have recently provided evidence that this is due to cells in the inferior colliculus (IC) responding to instances where multiple pulses are separated by short inter-pulse intervals. This is qualitatively similar to the facilitation mechanism described above, in that the effects of several closely spaced pulses combine to elicit temporal firing, although Hancock et al. suggested the operation of additional mechanisms such as the operation of low-voltage-activated potassium channels.

Rate discrimination tasks also potentially involve cues that do not require fine coding of inter-pulse intervals. Carlyon (1997) obtained pitch judgements for bandpass filtered pulse trains, where the probability, *p*, of a pulse occurring on each period could be less than 1. This allowed him to manipulate the mean pulse rate independently of the “common interval”, which was defined as the period of the intact pulse train (*p* = 1). He found that pitch judgements were affected by both the common interval and mean-rate cues. In our study, the jitter detection task relies only on the common interval cue, while the two cues can be used for the rate discrimination task. Because on average the CI listeners were able to perform the jitter task just as well as the NH listeners, our results indicate that the common interval cue is relatively preserved in electric hearing. The common effect of base rate across the two tasks in CI listeners indicates that the common interval cue is affected by base rate. Note that this does not mean that the mean-rate cue is not affected, but rather that it is not affected in the exact same way. More specifically, the variability observed for the jitter task across participants and conditions, and the lack of correlation between the jitter and the rate tasks suggest that the common interval cue might not be equally available to all CI listeners or to all electrodes. These listeners may thus be using different cues to perform the rate discrimination task.

One interesting implication of the above arguments concerns the identification of electrodes that do and do not convey useful temporal information. It is well-known that performance in rate discrimination tasks can differ across electrodes for the same subject (Kong et al. 2009; Carlyon and Deeks 2013; Ihlefeld et al. 2015), and our results extend this finding to the detection of temporal jitter. However, our results also show that the two tasks can show opposite effects for the two electrodes, for example for the first three CI listeners shown in Figure 2 for the 100-pps condition. Given recent interest in re-programming CIs based on performance in psychophysical tasks (e.g. Bierer 2010; Garadat et al. 2013), it would be interesting to determine which, if either, of the rate- and jitter-discrimination tasks produces performance that can be effectively used to guide electrode selection on a patient-by-patient basis.

## Footnotes

1 The slopes were obtained by refitting the LMM including only the significant effects in the model.

## References

[CR1] Bakeman R (2005). Recommended effect size statistics for repeated measures designs. Behav Res Methods.

[CR2] Bates D, Mächler M, Bolker B, Walker S (2015). Fitting linear mixed-effects models using lme4. J Stat Softw.

[CR3] Baumann U, Nobbe A (2004). Pulse rate discrimination with deeply inserted electrode arrays. Hear Res.

[CR4] Bierer JA (2010). Probing the electrode-neuron interface with focused cochlear implant stimulation. Trends Amplif.

[CR5] Bland JM, Altman DG (1995). Calculating correlation coefficients with repeated observations: Part 2—correlation between subjects. Br Med J.

[CR6] Bland JM, Altman DG (1995). Calculating correlation coefficients with repeated observations: part 1—correlation within subjects. Br Med J.

[CR7] Carlyon RP (1997). The effects of two temporal cues on pitch judgments. J Acoust Soc Am.

[CR8] Carlyon RP, Deeks JM (2002). Limitations on rate discrimination. J Acoust Soc Am.

[CR9] Carlyon RP, Deeks JM, Moore BCJ, Patterson RD, Winter IM (2013). Relationships between auditory nerve activity and temporal pitch perception in cochlear implant users. Basic aspects of hearing.

[CR10] Carlyon RP, Lynch C, Deeks JM (2010). Effect of stimulus level and place of stimulation on temporal pitch perception by cochlear implant users. J Acoust Soc Am.

[CR11] Core Team R (2015). R: a language and environment for statistical computing.

[CR12] Dobie RA, Dillier N (1985). Some aspects of temporal coding for single-channel electrical stimulation of the cochlea. Hear Res.

[CR13] Fox J, Weisberg S (2011). An R companion to applied regression, second.

[CR14] Garadat SN, Zwolan TA, Pfingst BE (2013). Using temporal modulation sensitivity to select stimulation sites for processor MAPs in cochlear implant listeners. Audiol Neurootol.

[CR15] Hancock KE, Chung Y, Delgutte B (2012). Neural ITD coding with bilateral cochlear implants: effect of binaurally coherent jitter. J Neurophysiol.

[CR16] Hochmair-Desoyer IJ, Hochmair ES, Burian K, Stiglbrunner HK (1983). Percepts from the Vienna cochlear prosthesis. Ann NY Acad Sci.

[CR17] Ihlefeld A, Carlyon RP, Kan A (2015). Limitations on monaural and binaural temporal processing in bilateral cochlear implant listeners. J Assoc Res Otolaryngol.

[CR18] Kong Y-Y, Carlyon RP (2010). Temporal pitch perception at high rates in cochlear implants. J Acoust Soc Am.

[CR19] Kong Y-Y, Deeks JM, Axon PR, Carlyon RP (2009). Limits of temporal pitch in cochlear implants. J Acoust Soc Am.

[CR20] Krumbholz K, Patterson RD, Pressnitzer D (2000). The lower limit of pitch as determined by rate discrimination. J Acoust Soc Am.

[CR21] Kuznetsova A, Brockhoff PB, Christensen RHB (2016) lmerTest: tests in linear mixed effects models

[CR22] Laback B, Majdak P (2008). Binaural jitter improves interaural time-difference sensitivity of cochlear implantees at high pulse rates. Proc Natl Acad Sci U S A.

[CR23] Lawrence MA (2015) ez: easy analysis and visualization of factorial experiments

[CR24] McKay CM, Carlyon RP (1999). Dual temporal pitch percepts from acoustic and electric amplitude-modulated pulse trains. J Acoust Soc Am.

[CR25] Moore BCJ, Carlyon RP, Plack CJ, Oxenham AJ, Fay RR, Popper AN (2005). Perception of pitch by people with cochlear hearing loss and by cochlear implant users. Pitch: neural coding and perception.

[CR26] Moore BCJ, Huss M, Vickers DA (2000). A test for the diagnosis of dead regions in the cochlea. Br J Audiol.

[CR27] Shackleton TM, Carlyon RP (1994). The role of resolved and unresolved harmonics in pitch perception and frequency modulation discrimination. J Acoust Soc Am.

[CR28] Shannon RV (1983). Multichannel electrical stimulation of the auditory nerve in man. I. Basic psychophysics. Hear Res.

[CR29] Stahl P, Macherey O, Meunier S, Roman S (2013) Rate discrimination at low pulse rate: comparison between normal-hearing and cochlear implants listeners and influence of intracochlear stimulation site. Lake Tahoe, CA 10.1121/1.4944564 27106306

[CR30] Studebaker GA (1985). A “rationalized” arcsine transform. J Speech Hear Res.

[CR31] Townshend B, Cotter N, Compernolle DV, White RL (1987). Pitch perception by cochlear implant subjects. J Acoust Soc Am.

[CR32] Tsuzaki M, Patterson RD, Palmer AR, Summerfield AQ, Meddis R (1998). Jitter detection: a brief review and some new experiments. Psychophysical and physiological advances in hearing: Proceedings of the 11th International Symposium on Hearing, Grantham, U.K., 1–6th August, 1997.

[CR33] van Wieringen A, Carlyon RP, Long CJ, Wouters J (2003). Pitch of amplitude-modulated irregular-rate stimuli in acoustic and electric hearing. J Acoust Soc Am.

